# Annuloplasty Ring Utilization in Mitral Valve Repair: A Real-World Snapshot of Device Selection and Early Outcomes

**DOI:** 10.3390/jcm15103711

**Published:** 2026-05-12

**Authors:** Andrzej Klapkowski, Aleksandra Stańska, Nikodem Ulatowski, Maciej Duda, Igor Tomczyk, Wojciech Karolak

**Affiliations:** 1 Department of Cardiac Surgery, Faculty of Medicine, Medical University of Gdańsk, ul. Skłodowskiej-Curie 3a, 80-210 Gdańsk, Poland; nikodem.ulatowski@gumed.edu.pl (N.U.); doodak@gmail.com (M.D.); i.tomczyk@gumed.edu.pl (I.T.); wkarolak@uck.gda.pl (W.K.); 2 Division of Quality of Life Research, Department of Psychology, Faculty of Health Sciences, Medical University of Gdańsk, ul. Skłodowskiej-Curie 3a, 80-210 Gdańsk, Poland; astanska@gumed.edu.pl

**Keywords:** mitral valve repair, annuloplasty ring, postoperative atrial fibrillation

## Abstract

**Background**: Annuloplasty is a key component of mitral valve repair, yet the impact of ring design on early outcomes remains unclear. **Methods**: This retrospective study included 153 consecutive patients undergoing mitral valve repair with annuloplasty. Ring designs were grouped into semi-rigid rings, semi-rigid bands, rigid rings, and other designs. The primary outcome was new-onset postoperative atrial fibrillation (AF). Secondary outcomes included early complications and repair durability based on follow-up echocardiography. **Results**: Postoperative AF occurred in 14.4% of patients and did not differ across ring design groups (*p* = 0.791). No independent predictors of AF were identified, although a longer aortic cross-clamp time showed a borderline association. Early outcomes were favorable, with 2.0% mortality and 5.9% rethoracotomy. Follow-up echocardiography was available in 79.7% of patients, with good or moderate repair observed in 95.9%. Echocardiographic repair failure occurred in 2.5% of patients. No clear association was observed between ring design and repair durability. **Conclusions**: In this real-world cohort, no significant association was detected between annuloplasty ring design and early postoperative AF or short-term outcomes. These findings should be interpreted cautiously, given the low event rate and small subgroup sizes.

## 1. Introduction

Mitral valve repair is the preferred surgical strategy for degenerative mitral regurgitation when feasible, offering durable valve competence with preservation of native valve apparatus [1,2]. Annuloplasty is a core component of contemporary repair, providing annular stabilization and supporting leaflet coaptation through the use of prosthetic rings or bands [3]. Despite broad adoption, ring selection remains heterogeneous across centers and surgeons, reflecting differences in valve pathology, annular geometry, repair philosophy, and device-specific design features (e.g., rigid rings, semi-rigid rings, and semi-rigid bands) [4,5].

Postoperative atrial fibrillation (AF) is one of the most common early complications after cardiac surgery and remains clinically relevant due to its association with prolonged hospitalization, thromboembolic risk, and resource utilization [6,7,8]. While patient-related factors (such as age and comorbidity burden) are well-recognized contributors to postoperative AF, the extent to which procedural choices during mitral repair, including annuloplasty ring platform, might relate to early AF remains less clear in routine practice [9,10]. Similarly, early safety outcomes such as rethoracotomy and in-hospital mortality are infrequent after isolated repair, but they provide an important pragmatic benchmark when describing real-world procedural cohorts.

Therefore, the aim of this study was to provide a real-world snapshot of annuloplasty ring utilization in mitral valve repair and to explore whether ring design categories show any association with early postoperative AF and short-term safety endpoints in a consecutive case series.

## 2. Methods

### 2.1. Study Design and Setting

This retrospective observational study summarizes real-world practice patterns and early outcomes in patients undergoing mitral valve repair with implantation of an annuloplasty ring. The cohort was derived from consecutive cases from a single-surgeon series captured in a routinely maintained clinical database. Data were extracted retrospectively for the purposes of the present analysis.

### 2.2. Patient Population

Adult patients undergoing mitral valve repair with annuloplasty ring implantation were eligible for this analysis. Ring-focused analyses were restricted to cases in which the implanted ring model could be classified into prespecified design categories. Patients undergoing mitral valve replacement were not considered in the present study.

### 2.3. Surgical Technique and Procedural Variables

All mitral valve repairs were performed through a minimally invasive right anterolateral minithoracotomy approach. In more recent cases, a totally endoscopic technique was used. Procedures were performed using standard contemporary techniques, with annuloplasty ring implantation as a core component of valve stabilization. Repair strategy was categorized into clinically interpretable technique groups:

- Isolated ring annuloplasty;
- Chordal replacement plus ring annuloplasty;
- Other repair variants like cleft closures, commissure closures, patch repairs.

Rare cases of redo procedures (*n* = 1) involving isolated chordal repair without additional ring implantation were retained in the dataset, as these patients had prior annuloplasty and were managed within the same clinical pathway.

Procedural variables collected for analysis included aortic cross-clamp time, cardioplegia strategy (Del Nido vs. HTK Brettschneider), and concomitant procedures, including tricuspid intervention and patent foramen ovale (PFO) closure.

### 2.4. Annuloplasty Ring Classification and Sizing

Annuloplasty rings were classified by model into named commercially available designs. For group-level analyses, ring models were consolidated into four clinically readable categories:

- Semi-rigid rings: Carpentier-Edwards Physio II and Medtronic SimuForm;
- Semi-rigid bands: Carpentier-Edwards Physio Flex;
- Rigid rings: Medtronic Profile;
- Other designs (rare models pooled to ensure stable cell counts, including, e.g., Medtronic Simulus and St Jude Saddle rings).

Annuloplasty ring size was recorded as the implanted labeled size and summarized descriptively; comparisons were performed to assess whether ring size distributions differed by postoperative atrial fibrillation status. For descriptive purposes, individual ring models are presented, while grouped categories were used for comparative analyses.

### 2.5. Outcomes

Postoperative atrial fibrillation (AF) was defined as new-onset AF documented by continuous ECG monitoring during the index hospitalization. Patients with a prior history of AF were excluded from the primary AF analysis. AF events were identified based on routine clinical monitoring and ECG recordings during hospitalization. No post-discharge AF events were included in the analysis. This definition is consistent with prior studies evaluating postoperative AF and its clinical impact [11]. Secondary early outcomes included early mortality and rethoracotomy. As an exploratory descriptive endpoint, early postoperative echocardiographic findings were summarized where available. Outcomes were assessed during the index hospitalization and/or early postoperative period as captured in the clinical database.

### 2.6. Ethics Statement and Consent to Participate

This study was a retrospective analysis of routinely collected, fully anonymized clinical data and involved no additional procedures or patient contact. The use of hospital data for research purposes was permitted at the institutional level. Due to the non-interventional retrospective design and the use of anonymized records, individual informed consent was waived.

### 2.7. Statistical Analysis

Categorical variables are presented as counts and percentages. Continuous variables are summarized as mean and standard deviation (SD). Associations between categorical variables were evaluated using the chi-square test or Fisher’s exact test, as appropriate.

To explore independent associations with postoperative atrial fibrillation, multivariable logistic regression analysis was performed, including a predefined set of clinically relevant covariates (age, sex, aortic cross-clamp time, and ring design).

A two-sided *p* value < 0.05 was considered statistically significant. Analyses were conducted using an available-case approach, with denominators varying across analyses depending on data completeness. Statistical analyses were performed using SPSS version 23 (IBM Corp., Armonk, NY, USA).

## 3. Results

### 3.1. Cohort and Repair Technique Profile

The study cohort comprised 153 patients undergoing mitral valve repair with annuloplasty ring implantation. The population included 58 women (37.9%) and 95 men (62.1%), with a mean age of 58.0 years (range 20–84 years).

Across analyses, denominators varied due to available case reporting. Ring-specific analyses were performed in the subset of patients with identifiable annuloplasty ring model information.

Among patients undergoing mitral valve repair, the predominant surgical strategy was chordal replacement combined with ring annuloplasty, whereas isolated ring annuloplasty and other repair variants were less frequent (Table 1).

Valve morphology was associated with the selected repair strategy (Pearson Chi-square *p* < 0.001). In patients with Barlow disease, chordal replacement combined with ring annuloplasty was used in the vast majority of cases (91.8%), whereas isolated annuloplasty and other techniques were rare. In fibroelastic deficiency (FED), combined repair also predominated (93.3%). A similar pattern was observed in FED+, where chordal replacement with ring annuloplasty was performed in 86.1% of cases.

In contrast, patients with functional mitral regurgitation (functional MR) were treated predominantly with isolated ring annuloplasty (95.7%), reflecting a different repair strategy focused primarily on annular remodeling.

These findings highlight clear differences in surgical approach across valve morphologies. However, given the small subgroup sizes and the high proportion of cells with low expected counts, these results should be interpreted with caution.

### 3.2. Annuloplasty Ring Model Utilization

A broad range of annuloplasty ring models was used. The most frequently implanted design was Carpentier-Edwards Physio II (56.6%), accounting for more than half of the implants. Other commonly used models included Medtronic Profile (15.1%), Medtronic SimuForm (13.8%), and Carpentier-Edwards Physio Flex (12.5%).

Rare individual designs, including Simulus, Saddle, and Sorin Memo 3D, were grouped as “other” to maintain stable cell counts for group-level analyses (Table 2).

### 3.3. Annuloplasty Ring Size Distribution

Annuloplasty ring sizes ranged from 26 to 40 mm, with a mean implanted size of 34.3 ± 4.3 mm. The most commonly used sizes were 40 mm (23.0%), 30 mm (17.1%), and 32 mm (14.5%), with the majority of implants falling within the 32–40 mm range (Table 3).

### 3.4. Ring Design and Postoperative Atrial Fibrillation

In the overall cohort of 153 patients undergoing mitral valve repair, new-onset postoperative atrial fibrillation occurred in 22 patients (14.4%).

AF rates varied across ring design groups but did not differ significantly between them (Pearson Chi-square *p* = 0.791) (Table 4).

In multivariable logistic regression analysis, adjusting for age, sex, aortic cross-clamp time, and ring design, no independent predictors of postoperative AF were identified. A trend toward increased AF risk was observed with longer aortic cross-clamp time (OR 1.02 per minute, 95% CI 1.00–1.03, *p* = 0.052), although this did not reach statistical significance. No significant association was observed for annuloplasty ring design, age, or sex.

Given the relatively low number of AF events and small subgroup sizes, these findings should be interpreted with caution.

### 3.5. Early Echocardiographic Results

Early postoperative echocardiography demonstrated excellent repair quality in the majority of patients. A good immediate echocardiographic result was observed in 147 patients (96.7%), whereas 5 patients (3.3%) had suboptimal results.

Across ring design groups, no significant differences in early echocardiographic outcomes were observed (Pearson Chi-square *p* = 0.735); however, these comparisons are limited by the very low number of non-optimal outcomes and small expected cell counts.

### 3.6. Early Postoperative Outcomes

Early adverse events were uncommon (Table 5).

Early mortality occurred in three patients (2.0%), rethoracotomy in nine patients (5.9%), and conversion to sternotomy in five patients (3.3%). Postoperative stroke occurred in four patients (2.6%), and permanent pacemaker implantation was required in five patients (3.3%).

### 3.7. Predictors of Blood Transfusion

In multivariable logistic regression analysis, older age, female sex, and lower preoperative hemoglobin levels were independently associated with an increased likelihood of perioperative blood transfusion. Each one-year increase in age was associated with a higher probability of transfusion (OR 1.03, 95% CI 1.01–1.06, *p* = 0.021), while higher hemoglobin levels were associated with a reduced risk (OR 0.70, 95% CI 0.52–0.94, *p* = 0.018). Female sex remained a strong predictor of transfusion requirement (OR 0.20, 95% CI 0.09–0.45, *p* < 0.001).

Aortic cross-clamp time was not significantly associated with transfusion in the adjusted model (*p* = 0.166).

Given the limited number of events relative to the number of covariates, the multivariable model should be interpreted as exploratory and potentially underpowered.

### 3.8. Follow-Up Echocardiographic Outcomes

Follow-up echocardiography was available in 122 patients (79.7%), while 31 patients (20.3%) did not have available follow-up data. Follow-up echocardiography was routinely scheduled approximately three months after surgery.

Among patients with available follow-up imaging, a good repair result was observed in 107 patients (87.7%), moderate residual regurgitation in 10 patients (8.2%), and poor results in 3 patients (2.5%). Two patients died during the follow-up period and were analyzed separately.

### 3.9. Durability of Mitral Valve Repair

Repair durability was assessed using follow-up echocardiographic findings.

A durable repair result, including both good and moderate outcomes, was observed in 117 of 122 patients (95.9%), whereas echocardiographic repair failure occurred in 3 patients (2.5%).

Two patients died during the follow-up period and were analyzed separately from echocardiographic outcomes.

Analysis of repair durability according to ring design was performed in patients with available ring classification (*n* = 119). In this subgroup, echocardiographic repair failure occurred in three patients (2.5%) (Table 6).

Across ring design groups, durability remained high, with no clear pattern suggesting an association between ring type and repair failure. Given the very low number of events and small subgroup sizes, these findings should be interpreted with caution.

### 3.10. Repair Technique and Durability

No significant association between surgical repair strategy and echocardiographic durability was observed (Pearson Chi-square *p* = 0.131). Numerically higher failure rates were observed in patients undergoing isolated annuloplasty and in those treated with alternative repair techniques, whereas combined chordal replacement and ring annuloplasty showed the lowest proportion of failure. However, given the very low number of failure events, these findings should be interpreted with caution.

### 3.11. Valve Morphology and Repair Durability

No meaningful association between valve morphology and repair durability was observed. Echocardiographic repair failure occurred in a very small number of patients, with isolated events noted in the FED+ and functional MR groups, while no failures were observed in patients with fibroelastic deficiency (FED) or Barlow disease.

## 4. Discussion

In this real-world cohort of mitral valve repair supported by annuloplasty, chordal replacement combined with ring annuloplasty was the dominant repair strategy [12,13]. This distribution aligns with contemporary reconstructive practice, where restoration of leaflet coaptation is frequently paired with annular stabilization rather than isolated annuloplasty alone [14]. A broad spectrum of annuloplasty ring models was used, with semi-rigid Physio designs and SimuForm accounting for the majority of implants and semi-rigid bands representing a smaller subset, consistent with pragmatic ring selection driven by valve anatomy, surgeon preference, and device availability in routine clinical practice.

Across analyses, annuloplasty ring design did not demonstrate a clear association with postoperative atrial fibrillation (AF). In unadjusted comparisons, AF rates showed only modest variation across ring categories without consistent separation between groups. Importantly, when the ring design group was included in multivariable models alongside clinical and procedural covariates, ring design was not an independent predictor of postoperative AF. In contrast, patient age emerged as the most stable correlate, supporting the interpretation that early postoperative AF is largely determined by patient substrate rather than the annuloplasty platform itself. This observation is consistent with prior literature identifying age and atrial substrate as key determinants of postoperative AF following mitral valve surgery.

Early adverse events in the ring-repair cohort were infrequent. Descriptive comparisons did not suggest meaningful differences in early mortality or rethoracotomy between semi-rigid bands and rigid or semi-rigid rings, nor across the broader ring design groups; however, the absolute number of events was small and limits inference. Accordingly, the absence of statistically significant differences should be interpreted as a lack of a strong signal rather than evidence of equivalence between ring platforms for early outcomes.

Interestingly, exploratory analyses of perioperative transfusion revealed that female sex was strongly associated with a higher probability of blood transfusion, together with lower baseline hemoglobin levels, older age, and longer aortic cross-clamp time. These findings are consistent with growing evidence suggesting that women undergoing cardiac surgery may experience higher rates of perioperative transfusion and less favorable early outcomes, potentially reflecting differences in baseline hemoglobin levels, body size, and perioperative physiology [15,16]. Although transfusion risk was not a primary focus of the present analysis, these observations highlight the importance of considering sex-specific factors when evaluating perioperative risk profiles in mitral valve surgery.

From a clinical standpoint, the present data support a pragmatic message: within routine mitral valve repair practice, no significant association was detected between ring selection and early postoperative AF or short-term safety endpoints in this dataset, while patient-related factors (notably age and baseline clinical profile) appear more relevant for early postoperative risk. These findings should be interpreted within the broader context of the complexity and heterogeneity of mitral valve disease and evolving treatment strategies, as highlighted in the recent literature [17,18]. Future studies with larger samples and longer follow-up should prioritize outcomes where ring design may plausibly exert a stronger effect, including echocardiographic durability, recurrent regurgitation, ventricular remodeling metrics, and functional recovery. Additionally, richer phenotyping of valve pathology and repair complexity may help clarify whether specific ring platforms confer advantages in well-defined anatomical subsets.

### Study Limitations

This study has several limitations that should be acknowledged. First, the analysis was based on a retrospective observational cohort derived from a single-surgeon experience, which may limit generalizability and introduce potential selection bias. Second, although the overall sample size was moderate, some annuloplasty ring categories included relatively few patients, restricting statistical power for comparisons between individual device types. Third, the number of early adverse events was small, which limits the ability to detect subtle differences in outcomes between ring platforms. Fourth, follow-up echocardiographic data were not available for all patients, primarily because a proportion of individuals did not attend scheduled follow-up visits. Finally, the study focused primarily on early postoperative outcomes, and longer-term follow-up will be required to determine whether annuloplasty ring design influences repair durability, recurrent regurgitation, or ventricular remodeling over time.

## 5. Conclusions

In this real-world series of mitral valve repairs with annuloplasty, ring design was not independently associated with postoperative atrial fibrillation or early safety endpoints, whereas patient age showed the most consistent relationship with postoperative AF. Female sex was associated with a higher probability of perioperative blood transfusion, highlighting the potential importance of sex-related differences in perioperative risk. Larger cohorts and longer follow-up are warranted to determine whether ring selection influences longer-term repair durability and echocardiographic outcomes.

## Figures and Tables

**Table 1:** Mitral valve repair technique category.

Repair Technique Category	*n*	% (Valid)
Ring annuloplasty only	27	17.6
Chordal replacement only	1	0.7
Chordal replacement + ring annuloplasty	116	75.8
Other repair techniques	9	5.9

Valve morphology and repair strategy.

**Table 2:** Annuloplasty ring model distribution.

Annuloplasty Ring Model	*n*	% (Valid)
Carpentier-Edwards Physio II	86	56.6
Medtronic Profile	23	15.1
Medtronic SimuForm	21	13.8
Carpentier-Edwards Physio Flex	19	12.5
Other designs (Simulus, Saddle)	2	2.0

**Table 3:** Annuloplasty ring size distribution.

Ring Size (mm)	*n*	% (Valid)
26	2	1.3
28	17	11.2
30	26	17.1
32	22	14.5
34	14	9.2
36	20	13.2
38	16	10.5
40	35	23.0

**Table 4:** Postoperative atrial fibrillation according to ring design.

Ring Design Group	AF (*n*)	No AF (*n*)	Total (*n*)
Physio II	13	73	86
Physio Flex	3	16	19
Profile	5	18	23
SimuForm	1	20	21
Other designs	0	3	3

**Table 5:** Early postoperative outcomes.

Outcome	*n* (%)
Early mortality	3 (2.0%)
Rethoracotomy for bleeding	9 (5.9%)
Conversion to sternotomy	5 (3.3%)
Postoperative stroke	4 (2.6%)
Permanent pacemaker implantation	5 (3.3%)

**Table 6:** Repair durability according to ring design.

Ring Design	Durable Repair (*n*)	Repair Failure (*n*)	Total
Physio II	71	1	72
Physio Flex	6	0	6
Profile	18	1	19
SimuForm	20	0	20
Other designs	1	1	2

## Data Availability

The data presented in this study are available on request from the corresponding author. The data are not publicly available due to privacy and ethical restrictions related to clinical patient records.
